# Mitochondrial dysfunction in the bovine mammary gland: regulatory mechanisms and therapeutic strategies

**DOI:** 10.1186/s40104-026-01361-7

**Published:** 2026-03-18

**Authors:** Taiyu Shen, Ming Li, Xudong Sun, Zhaoju Deng, Hailey Pitts, Derek Nolan, Juan J. Loor, Chuang Xu

**Affiliations:** 1 National Key Laboratory of Veterinary Public Health and Safety, College of Veterinary Medicine, China Agricultural University, Beijing, 100193 China; 2 Heilongjiang Provincial Key Laboratory of Prevention and Control of Bovine Diseases, College of Animal Science and Veterinary Medicine, Heilongjiang Bayi Agricultural University, No. 5 Xinyang Road, Daqing, Heilongjiang Province 163319 China; 3 Department of Animal Sciences and Division of Nutritional Sciences, University of Illinois, Urbana, 61801 USA

**Keywords:** Apoptosis, Bovine, Innate immunity, Mammary gland homeostasis, Reactive oxygen species

## Abstract

Periparturient metabolic stress or pathogen infections leading to inflammation reduce milk yield and cause mammary dysfunction, thus, causing severe economic losses to dairy farming. As the primary organelle for cellular energy production, calcium regulation, cell death, and metabolism, mitochondrial homeostasis plays a crucial role in maintaining normal mammary gland function. This review focuses on the regulatory mechanisms of mitochondrial homeostasis and the effect of mitochondrial dysfunction on homeostatic mechanisms in bovine mammary gland. Furthermore, we summarize several potential therapeutic strategies targeting mitochondrial metabolic dysfunction to alleviate mammary tissue damage.

## Introduction

Achieving high rates of milk synthesis to help meet the growing demand for milk and dairy products requires high-genetic merit dairy cows to use a greater portion of absorbed nutrients to synthesize milk, hence, increase their productive efficiency [1]. At the onset of lactation, the high metabolic rate of the mammary gland directs the use of nutrients from the circulation towards milk synthesis [2]. Although there is an estimated 22% increase in the energy requirements during early lactation relative to late-lactation [3], the mammary gland absorbs 65%−84% of total metabolizable energy, which is almost 3 times the maintenance energy requirement of the animal [4]. Thus, high-genetic merit dairy cows maintaining lactogenic function and the numbers of mammary epithelial cells to cope with these high rates of milk synthesis is a crucial physiological undertaking.

Mitochondria within mammary epithelial cells are pivotal intracellular organelles for energy generation via the tricarboxylic acid cycle and oxidative phosphorylation (OXPHOS), processes that are intimately involved with milk synthesis [5, 6]. In response to the onset of lactation, the energy demands of the mammary gland are sustained in part by upregulation of OXPHOS and mitochondrial biogenesis. Indeed, between d 2 to 8 of lactation in mice, an increase in the number and size of mitochondria as well as increased ATP production have been reported [7]. In a similar fashion, the activity of the electron transport system complexes, production of ATP, and number of mitochondria increase in the mammary gland of dairy cows [8]. These processes respond further to higher milking frequency and allow for greater milk yield [9]. Besides the production of ATP, the mitochondria can produce reactive oxygen species (ROS), can act as storage of calcium ions, and can trigger programmed cell death [10–12]. Clearly, available studies demonstrate that mitochondrial function is essential for secretory capacity and activity of mammary epithelial cells in dairy cows.

Mitochondrial homeostasis in mammary epithelial cells of dairy cows undergoes serious metabolic challenges in early lactation, largely due to the gradual state of negative energy balance (NEB) occurring around parturition which drives the elevation in circulating concentrations of free fatty acids (FFA) [13, 14]. Although FFA uptake can contribute to milk fat synthesis and potentially ATP production in mammary epithelial cells of dairy cows, in large amounts they can induce mitochondrial dysfunction through the increase in ROS production, decreased mitochondrial DNA (mtDNA) copy number, and decreased ATP synthesis and mitochondrial numbers [11]. These events all contribute to the decrease of secretory capacity and number of mammary epithelial cells [15, 16]. In addition to metabolic challenges, pathogenic microorganisms and their metabolites can induce mitochondrial dysfunction of mammary epithelial cells. For example, Gao et al. [17] reported that *Staphylococcus aureus* resulted in mitochondrial damage and increased generation of mitochondrial ROS in mammary epithelial cells. Lipopolysaccharide (LPS), released by gram-negative bacteria, disrupts mitochondrial calcium ion homeostasis, induces mitochondrial damage, elevates mitochondrial ROS concentrations and results in apoptosis of mammary epithelial cells in dairy cows [10, 18]. Clearly, a multitude of factors can disrupt mitochondrial homeostasis, which contributes to the dysfunction of mammary epithelial cells in dairy cows.

In this review, we summarize research findings focused on the regulation of mitochondria and the effect of mitochondrial dysfunction on homeostasis of the mammary gland in dairy cows. We also discuss how new insights into potential therapies targeting mitochondria can help restore homeostasis of the mammary gland.

## Regulation of mitochondrial homeostasis

Mitochondria serve as the central hub of energy metabolism in bovine mammary epithelial cells (bMECs), supplying the necessary ATP for the synthesis of milk fat, milk protein, and lactose [19]. Beyond energy production, mitochondria also play vital roles in redox balance, calcium homeostasis, lipid metabolism, programmed cell death, and intracellular signaling. During lactation, bMECs enter a highly active synthetic state, exhibiting increased dependency on mitochondrial function and metabolic regulation [20]. Thus, maintaining mitochondrial integrity in terms of quantity, structure, and function is crucial for sustaining lactation performance. Although high-genetic merit dairy cows may encounter NEB and mitochondrial stress during early lactation, this section of the review focuses specifically on the core regulatory mechanisms that maintain mitochondrial homeostasis in bMECs. These include mitochondrial dynamics (fusion and fission), mitochondrial quality control including mitophagy and the ubiquitin–proteasome system (UPS), mitochondrial biogenesis, calcium signaling, and energy-sensing signaling pathways centered around AMP-activated protein kinase (AMPK). Rather than operating independently, these mechanisms are intricately interconnected, forming a coordinated and multi-layered network of mitochondrial quality control. This section aims to systematically discuss the specific roles and interrelationships of these pathways in bMECs, thereby providing a theoretical foundation for further understanding of mitochondrial homeostasis in the bovine mammary gland.

### Mitochondrial biogenesis

Mitochondrial biogenesis is a critical mechanism for maintaining both the quantity and quality of functional mitochondria. It refers to the process by which new mitochondrial components are synthesized and assembled into functional organelles under the coordinated regulation of nuclear-and mitochondrial-encoded genes [21]. The central regulatory factor of this process is peroxisome proliferator-activated receptor gamma coactivator 1-alpha (PGC-1α or *PPARGC1A*), a transcriptional coactivator that is activated by various stress signals (e.g., nutrient deprivation, hypoxia, oxidative stress, endoplasmic reticulum (ER) stress). Although PGC-1α is well-known to be central for mitochondrial function in tissues with oxidative metabolism capacity, the PGC-1β isotype also is recognized as an important co-activator of transcriptional events, and in innate immune cells is essential for mitochondrial metabolism [22]. Once activated, the PGC-1α and PGC-1β translocate into the nucleus and in the case of PGC-1α it promotes the expression of nuclear respiratory factor 1 and 2 (NRF1 and NRF2), as well as mitochondrial transcription factor A (TFAM), thereby enhancing mtDNA replication and transcription [21]. The activity of PGC-1α is regulated by multiple signaling pathways, including cAMP responsive element binding proteins (CREB) [23], sirtuin (SIRT) 1, AMPK, and protein modifications [24].

The first evaluation of a role for PGC-1α and PGC-1β in the mammary gland of high-genetic merit Holstein cows was published in 2008, where it was demonstrated that mRNA abundance for both genes (and especially *PPARGC1A*) was markedly upregulated during the lactation cycle relative to two weeks prior to parturition [25]. The profiles of these two genes were associated with the profiles of several genes associated with milk fat synthesis, and also with the trend in milk yield during the entire lactation, underscoring important biological associations between mitochondria and mammary cell function. Mechanistically, the upregulation of CREB1 and CREB3 in the mammary gland during lactation [26] could serve to activate transcription of *PGC-1α*, thus, aiding the function of this protein in the context of mitochondrial biogenesis. The regulation of CREB proteins is itself complex, for instance several growth factors and inflammatory signals can induce CREB expression [27], but specific mechanisms in the bovine mammary gland are not well studied.

The demand for energy to synthesize lactose, protein, and lipid in the mammary gland increases significantly after parturition, leading to the transcriptional upregulation of several mitochondrial-related biological processes [26, 28] such as formation of the outer and inner membrane, respiratory chain complexes, matrix, proton-transporting ATP synthase complex, and an overall increase in mitochondrial biogenesis [29]. A correlation between *PPARGC1A* mRNA abundance and mtDNA copy number as a function of stage of lactation was reported recently, and underscored that higher milk yield in early- relative to late-lactation is partly sustained by enhanced mitochondrial function [3]. Higher ATP production has been observed in mouse mammary glands during early lactation, and was attributed to the overall increase in mtDNA copy number, mitochondrial size, and mitochondrial density [30]. Together, the available data underscore the biological associations between mitochondria, ATP production, and mammary cell lactogenic capacity.

Recent studies in murine mammary epithelial cells (MECs) have revealed a novel regulatory network of mammary cell mitochondrial biogenesis, where arginine activates mechanistic target of rapamycin (mTOR) complex 1 (mTORC1) via CASTOR1 and promotes mitochondrial biogenesis through the mTORC1/PGC-1α/NRF1 axis thereby supporting mitochondrial homeostasis, ATP synthesis, and redox balance [31]. An in vitro study with murine MECs revealed that reductions in energy availability led to inhibition of milk fat and protein synthesis, while the AMPK signaling pathway was activated to inhibit PGC-1α acetylation thereby enhancing fatty acid oxidation [32]. It is unclear if these exact mechanisms also function in the bovine mammary gland because arginine reduces p-AMPK and enhances p-mTOR/total mTOR in isolated MECs without affecting CASTOR1 mRNA or protein abundance [33]. However, as reported in the mouse, the fact that PGC-1α, mTORC1, and several components of the protein synthesis machinery and functional mitochondria are upregulated in mammary tissue after parturition and through most of lactation [25, 26, 34] is suggestive of novel regulatory networks.

In the context of cellular energy metabolism, it is possible that an elevated AMP/ATP ratio may lead to excessive AMPK activation (as observed in liver from ketotic dairy cows; [35]), potentially resulting in mitochondrial overload and a ROS burst. A link between mammary metabolism and AMPK activation during lactation in Holstein cows was confirmed by the upregulation of *AMPK* mRNA abundance after parturition and through the first 60 d postpartum [36]. Mitochondrial biogenesis and autophagy also exhibit spatiotemporal coordination in the bovine mammary gland [36], where the increase in metabolic rate (and potentially oxidative and ER stress) after parturition could induce mitochondrial damage and inflammation in mammary epithelial cells, necessitating a concomitant upregulation of autophagic activity to remove excess or dysfunctional mitochondria (or other organelles or molecules) and modulate inflammation. This is exemplified by the marked activation of genes associated with regulation of the I-κB kinase/NF-κB cascade, DNA damage response, release of cytochrome c from mitochondria, ubiquitination, and ER stress [36].

As reported in vitro with bMECs incubated with the protein chemerin, which promoted autophagy and apoptosis, a disruption of the balance between autophagy and apoptosis could lead to mitochondrial fragmentation (i.e., fission) and reduced milk protein secretion efficiency [37]. Whether such an event occurs in the bovine mammary gland in vivo is doubtful because the abundance of chemerin (also known as RARRES2) was markedly downregulated between late prepartum and peak lactation [26]. In addition, other than a marked upregulation at d −15 and 1 relative to d −30 around parturition, several genes associated with mitochondrial fission remained downregulated through day 60 postpartum [26], which agrees with the concept that states of low ATP demands enhance mitochondrial fragmentation [38]. At least judging from mRNA abundance data [36], a return to an activated state of mitochondrial fission (and fusion) was detected at 120 to 300 d postpartum, suggesting that the normal decrease in synthetic capacity of the MECs after mid-lactation also reduces the need for cellular ATP production.

From a biological standpoint, and similar to non-ruminants, it is possible that the sudden need for ATP close to parturition to synthesize colostrum and shortly after parturition to synthesize mature milk requires the engagement of mitochondrial fission and fusion, as a short-term adaptation to a changing environmental state [38]. As lactation progresses, dairy cows are able to consume greater amounts of feed and enhance the number of substrates available for gluconeogenesis and also for ATP synthesis, e.g., acetate and hydroxybutyrate, likely reducing the need for an activation of mitochondrial fission and fusion.

Epigenetic regulation plays an emerging role in mitochondrial biogenesis. Dynamic DNA methylation changes serve as molecular switches for mitochondrial proliferation. In ovine mammary epithelial cells, miR-148a targets both DNMT1 and the 3′-UTR of PGC-1α, reducing their expression and thereby facilitating demethylation and transcriptional upregulation of PGC-1α. This process significantly enhances the expression of mitochondrial genes and cellular bioenergetic capacity [39]. Histone acetylation and methylation also critically influence chromatin accessibility and transcription factor recruitment. EZH2, a demethylase targeting H3K27, and p300, an H3K9 acetyltransferase, modulate the expression of NRF1 and TFAM in mammary epithelial cells, respectively, thereby affecting mitochondrial biogenesis [40]. PGC-1α itself is subject to histone-like post-translational modifications; for instance, SENP1, a SUMO-specific protease, deSUMOylates PGC-1α, enhancing its interaction with transcription factors such as ERRα and PPARγ, thus, boosting downstream mitochondrial gene expression and biogenesis [41]. These findings indicate that mitochondrial biogenesis is not solely governed by classical transcriptional networks but is also deeply intertwined with the metabolic microenvironment and epigenetic signaling pathways.

The functional crosstalk between mitochondrial biogenesis and the UPS deserves attention. As the "master switch" of mitochondrial biogenesis, PGC-1α’s nuclear stability is tightly regulated by UPS. It has a short half-life of approximately 0.3 h in the nucleus and undergoes rapid degradation via ubiquitination and proteasomal processing [42]. The E3 ubiquitin ligase FBXW7 has been shown to recognize PGC-1α and promote its ubiquitin-mediated degradation, thereby negatively regulating mitochondrial biogenesis [43]. Conversely, in mammary epithelial cells, the deubiquitinase USP22 stabilizes PGC-1α and its coactivators by removing ubiquitin chains, thereby supporting efficient mitochondrial biogenesis and oxidative metabolism to meet the energy demands during mammary gland development [44].

This dynamic balance between deubiquitination and re-ubiquitination may act as a finely tuned mechanism that modulates mitochondrial quantity and function in bMECs during lactation or differentiation.

In summary, mitochondrial biogenesis acts as a compensatory mechanism that, along with mitochondrial “clearance” (via autophagy and UPS) and “repair” (via molecular chaperones), forms a multilayered network regulating mitochondrial homeostasis. In the following section, we will discuss how AMPK, as a key energy sensor, orchestrates these mechanisms to facilitate metabolic reprogramming in bMECs.

### Mitochondrial dynamics

Mitochondria exhibit high plasticity, with their dynamic changes primarily manifested through continuous fusion and fission. This structural remodeling mechanism serves as the foundation for bMECs to adapt to fluctuations in metabolic demand (e.g., need for ATP), ER stress, oxidative stress, and maintenance of cellular homeostasis [45]. Mitochondrial fusion, regulated by Mitofusin 1/2 (MFN1/2) for outer membrane integration and by optic atrophy 1 (OPA1) for inner membrane maintenance, facilitates mitochondrial genome integration, preservation of membrane potential, and enhanced capacity for OXPHOS [46, 47]. Research demonstrated that the GTP hydrolysis mechanism of MFN1 mediates mitochondrial outer membrane tethering and fusion through conformational changes, with structural analyses revealing that GTP-bound MFN1 drives membrane fusion via helical domain rearrangement [48].

The first report of temporal changes in mRNA abundance of genes associated with mitochondrial fission and fusion in the mammary gland of high-producing Holstein cows revealed that both processes are markedly activated close to parturition and at calving relative to one month prior to parturition [36]. The temporal mRNA abundance of *MFN1* decreased markedly between d −15 prepartum to d 120 postpartum, which coincided with a marked downregulation of fusion and fission [26]. Whether a shortfall in the availability of GTP is associated with MFN1 function in bMECs is unclear. However, the fact that the mRNA abundance for the rate-limiting enzyme in de novo GTP synthesis (inosine monophosphate dehydrogenase, IMPDH1) was consistently upregulated during lactation [26] suggests that the supply of GTP is not a limiting factor in MFN1 activity during the normal course of lactation. In mammary epithelial cells, a balanced mitochondrial fission–fusion dynamics ensures the control of energy metabolism to meet the high lactation-associated ATP demands [49]. It is noteworthy that MFN2 regulates multiple processes beyond fusion, including ER tethering for calcium ion flux modulation. Specifically, in bMECs and mice, MFN2-mediated mitochondria-associated ER membranes (MAMs) influence ER stress responses through calcium homeostasis regulation [50, 51]. This raises the possibility that the MFN2 may be involved in the pathogenesis of bovine mastitis, though direct evidence in mastitis models remains to be established.

### Mitochondrial quality control

In bMECs, mitochondrial turnover becomes particularly crucial due to the significantly increased metabolic load during lactation [18]. As mitochondria serve as the primary source of ROS, mitochondrial proteins are exposed to high concentrations of ROS and its damaging effects. Thus, the restoration of stressed mitochondria and renewal of damaged mitochondria constitute critical steps in maintaining mammary epithelial cell bioenergetics [52]. Studies in human and murine models have identified three primary pathways for maintaining mitochondrial integrity depending on the severity of the damage.

First, mitophagy represents a highly selective form of autophagy responsible for identifying and removing damaged or senescent mitochondria, thereby maintaining the overall stability of the mitochondrial population within cells [53]. In dairy cows with periparturient ketosis, elevated β-hydroxybutyrate (BHB) concentrations impair lysosomal acidification and disrupt autophagosome-lysosome fusion, leading to the accumulation of dysfunctional mitochondria and cytoplasmic release of mtDNA. This mtDNA leakage activates the cyclic GMP-AMP synthase (cGAS) stimulator of interferon genes (STING) pathway, triggering inflammation in bMECs [54]. Pharmacological interventions in vitro such as rapamycin or mitophagy activators can alleviate this pathological progression in the bMECs [54]. The PINK1-Parkin pathway constitutes the core molecular mechanism regulating mitophagy, with its operational framework having been extensively characterized in non-ruminants across diverse physiological and pathological contexts. Upon mitochondrial membrane potential (ΔΨm) dissipation, PINK1 (PTEN-induced putative kinase 1) stabilizes on the outer mitochondrial membrane and phosphorylates both ubiquitin and Parkin at Ser65 residues. This phosphorylation cascade subsequently recruits cytosolic Parkin (an E3 ubiquitin ligase) to mitochondrial surfaces, where it initiates ubiquitination modifications and orchestrates the recruitment of autophagy receptor proteins (e.g., OPTN, NDP52). These molecular events ultimately facilitate the clearance of damaged mitochondria via the autophagic-lysosomal system [55, 56].

The detailed biochemical steps and regulatory mechanisms underlying ubiquitin conjugation to damaged mitochondria via the PINK1-Parkin axis are comprehensively described elsewhere in a specialized review [57]. Although there is transcriptional activation of the inflammatory I-κB kinase and NF-κB cascade and the complement system as lactation progresses in the mammary gland of clinically-healthy Holstein cows, the mRNA abundance of *PINK1* and pathways associated with autophagy and macroautophagy are gradually downregulated [26], suggesting that during a normal lactation there are mechanisms that prevent excessive damage of mitochondria. This is an intriguing idea because autophagy adaptors encoded by genes such as Tax1 binding protein 1 (*TAX1BP1*) which inhibits inflammation, genes like fission 1 (*FIS1*) and ubiquitin protein ligase 1 (*MUL1*) which regulate mitochondrial morphology/apoptosis and fission, and genes associated with protein ubiquitination are markedly upregulated after parturition through at least 60 d in milk [26]. One potential biological role for the upregulation of MUL1 during lactation besides autophagy and fission is to ubiquitinate proteins that can sustain the NF-κB inflammatory cascade [58]. As such, MUL1 could contribute to the control of inflammation and work along a number of antioxidant proteins with important functions in controlling oxidative stress in mammary tissue after parturition [59].

In contrast to what occurs during the normal functioning of the mammary gland, infections caused by microorganisms such as *Staphylococcus* trigger a PINK1/Parkin-mediated mitophagy response that reduces mitochondrial ROS (mtROS) levels, thereby effectively suppressing NLRP3 inflammasome assembly and activation of the NF-κB signaling pathway [60]. However, excessive activation of mitophagy may elicit pathological sequelae: in MEC models of heat stress (HS)-associated disorders, suppression of PINK1/Parkin-mediated mitophagy alleviates the HS-induced impairment in mitochondrial homeostasis. Thus, mitochondrial homeostasis ensures ATP synthesis and maintains cellular redox homeostasis in HS-challenged MECs, thereby further mitigating HS-induced cellular damage [31]. This, and other mechanisms discussed in preceding sections of this review, underscore the necessity for dynamic equilibrium between autophagic activity and mitochondrial biogenesis. Furthermore, the UPS indirectly modulates mitophagy progression through degradation of autophagy-related proteins such as p62. Parkin, serving as a molecular bridge connecting UPS and mitophagy, orchestrates a dual-layer quality control mechanism: its ubiquitination signaling function not only facilitates autophagosome formation by recruiting receptor proteins (e.g., NBR1, NDP52) [61], but also guides directly the proteasomal recognition and degradation of damaged proteins exposed on mitochondrial membranes [62].

A second regulatory mechanism involves the formation of mitochondrial-derived vesicles (MDV), a vesicular trafficking system that facilitates the removal of oxidized and/or damaged proteins and lipids via the lysosome, or the delivery of cargo to the peroxisome [63]. This system has been demonstrated to play a critical role in mitochondrial quality control in non-ruminant models [64, 65]. The bovine mammary gland expresses genes for small GTPases (*RAB7B*) and toll interacting protein (*TOLLIP*) that help control fusion with lysosomes and endolysosomal sorting as part of the MDV targeting process [63]. There are also MDV that target the peroxisome and contribute to its biogenesis, including the delivery of MUL1 to new peroxisomes which was the first described MDV transport pathway. Although there are several peroxisomal biogenesis factors that are upregulated in mammary tissue after parturition [26], it is unclear if any of these are delivered as MDV. Based on the available data, it is possible that MUL1 is delivered via the MDV which then stimulates the fusion of new peroxisomes and the upregulation of peroxisomal biogenesis genes (PEX).

The third quality control mechanism entails the degradation of misfolded mitochondrial proteins by AAA proteases localized within the mitochondrial inner membrane and matrix [66]. It is noteworthy that significant knowledge gaps persist regarding the precise regulatory networks governing mitochondrial quality control in bMECs. Although formation and transport of MDV and AAA proteases in the context of mitochondrial homeostasis have gained experimental support in human and murine models, cautious validation remains imperative when extrapolating these findings to the bovine-specific metabolic milieu. Particularly considering the unique metabolic signatures of lactating dairy cows, including but not limited to intense lipid biosynthesis, distinctive glucose metabolic routing, and molecular regulatory networks governing milk component secretion. Future investigations require the development of bMECs-optimized experimental models that enable systematic investigation of species-specific regulatory nodes in MDV biogenesis/trafficking as well as dynamic adaptation mechanisms of mitochondrial protease systems under sustained lactational stress. Such research will not only refine theoretical frameworks in ruminant cell biology but also provide molecularly precise targets for enhancing bovine mammary health.

### Mitochondrial calcium (Ca^2^⁺) homeostasis

Mitochondrial Ca^2+^ signaling plays a central regulatory role in maintaining cellular energy metabolism, oxidative and ER stress responses, and programmed cell death in bMECs, particularly under pathological stimuli such as inflammation and HS [10]. Calcium movement into the mitochondria can occur via voltage-dependent anion-selective channels (VDAC), mitochondrial calcium uniporters (MCUR), rapid mitochondrial uptake, ryanodine receptors, or Na⁺/Ca^2+^ exchanger (SLC8A1-3) [67]. The MUC, highly selective for the transport of Ca^2+^, is composed of several regulatory proteins including MCUR1 and its paralog CCDC90B and is the main route whereby Ca^2+^ flows into the mitochondria [68]. In the context of Ca^2+^ in the bovine mammary gland, it is noteworthy that several pathways associated with Ca^2+^ signaling and transport were markedly upregulated after parturition and through late-lactation [26]. Although none of these pathways pertain to mitochondrial metabolism or function, the fact that mRNA abundance of *CCDC90B* was nearly twofold greater at 120 d in milk relative to the dry period suggests a potential association with MUC in the influx of Ca^2+^ into the mitochondria.

Under environmental stress or during pathogenic infections, recent data indicate that mitochondrial Ca^2+^ homeostasis in bMECs is disrupted, triggering mitochondrial dysfunction and apoptosis. Stimulation of bMECs with LPS led to elevated MCUR1 expression, leading to mitochondrial Ca^2+^ overload, increased ROS levels, reduced ΔΨm, and exacerbated apoptosis. Notably, *MCUR1* knockdown partially reversed this cascade of events, suggesting its critical involvement in the immune response and repair mechanisms through regulation of mitochondrial Ca^2+^ homeostasis [10]. Conditions like HS disrupt mitochondrial network dynamics in bMECs and are accompanied by aberrant Ca^2+^ influx into mitochondria, impaired OXPHOS and ATP production. This Ca^2+^ overload further amplifies intracellular ROS accumulation and cytochrome c release, culminating in apoptotic pathway activation [12]. Bacterial infections also induce analogous mitochondrial Ca^2+^ dysregulation. For instance, Klebsiella pneumoniae-infected bMECs exhibit significantly elevated mitochondrial Ca^2+^ concentrations concurrent with ΔΨm collapse and ROS surge, implying a potential role of Ca^2+^ homeostasis disruption in mediating mitochondrial dysfunction during bacterial infection [69].

Despite the potential for aberrant mitochondrial Ca^2+^ metabolism under pathogen and environmental conditions, it is noteworthy that genes associated with the release of cytochrome c from mitochondria in mammary tissue from clinically-healthy Holstein cows are markedly upregulated at d −15 and throughout lactation relative to d −30 prepartum [26]. This suggests that, beyond its crucial role in energy metabolism, the mitochondria are intimately involved in mammary cell function, potentially by controlling the rate of cellular apoptosis which is an important determinant of mammary cell turnover during lactation [70].

As a common metabolic disorder in high-producing dairy cows during the peripartum period, hypocalcemia induces compensatory imbalance in the body's calcium transport regulation. This compensatory regulatory disorder can further disrupt the intracellular calcium homeostasis of bMECs, thereby triggering mitochondrial calcium overload. On one hand, mitochondrial calcium overload impairs the integrity of the mitochondrial membrane structure, leading to a decrease ΔΨm; on the other hand, it activates oxidative stress responses, promoting the massive production of ROS. However, current research on abnormal mammary mitochondrial metabolism in hypocalcemic dairy cows is still inadequate, and its specific regulatory mechanism needs further verification and confirmation.

Overall, the available data underscore that mitochondrial Ca^2+^ signaling is pivotal in orchestrating bMECs adaptations to the onset of lactation and exogenous stressors. Disrupted Ca^2+^ homeostasis not only compromises mitochondrial bioenergetics but under normal conditions can activate cell death pathways that participate in the control of mammary cell turnover. Under stress conditions, aberrant Ca^2+^ signaling can contribute to inflammation and damage of mammary tissue. In this context, evaluation of therapeutic strategies targeting mitochondrial Ca^2+^ channels or regulatory factors may yield novel molecular interventions for bovine mastitis and related pathologies.

### AMPK and beyond

In bMECs, AMPK acts as a critical energy sensor that monitors the intracellular ATP/AMP ratio to regulate cellular metabolism and mitochondrial homeostasis [71]. AMPK not only functions as a metabolic “switch” but also serves as a central hub coordinating nutrient sensing, lipid metabolism, mitochondrial function, and autophagy enabling the cells to adapt to metabolic fluctuations and sustain mammary gland health and lactation efficiency [25, 57]. The fact that *AMPK* mRNA abundance in bovine mammary tissue is markedly upregulated through at least 60 d postpartum [36] underscores the importance of this signaling network as a key regulator of bMECs homeostasis. It represents a promising target for alleviating nutritional stress and enhancing mammary gland function.

The first longitudinal profiling of mTOR and mammary protein regulatory mechanisms across lactation was reported by Bionaz and Loor [34], providing foundational insight into the dynamic regulation of these pathways in vivo. Using serial bovine mammary biopsies from late-pregnancy through the end of lactation, their study revealed that the expression of key genes involved in mTOR signaling (*FRAP1*, *EIF4EBP2*, *GSK3A*, *TSC1*), amino acid and glucose transporters, and insulin signaling components markedly increased during lactation. These findings highlighted the central role of nutrient sensing and insulin-mediated mTOR activation in supporting milk protein synthesis. Despite the upregulation of certain negative regulators such as EIF4EBP2 and TSC1, the translational machinery remained active, likely due to insulin overriding inhibitory signals via the mTOR pathway. Importantly, no enhanced AMPK signaling was observed, suggesting that under the normal physiological conditions that characterize lactation, AMPK may not be essential for regulating milk protein synthesis.

Under metabolic stress conditions, AMPK exerts an antagonistic effect on the mTOR signaling pathway. Studies have shown that nutrient deprivation in bMECs markedly increases the AMP/ATP ratio, thereby activating AMPK signaling. This activation leads to phosphorylation of the downstream effector TSC2, inhibition of Rheb activity, and subsequent suppression of mTORC1-mediated protein synthesis and energy consumption. Inhibition of mTORC1 further reduces phosphorylation of S6K1 and 4E-BP1, thereby downregulating the expression of mitochondria-associated proteins and compromising cellular growth and proliferation support [72]. AMPK-mediated suppression of mTOR facilitates metabolic reprogramming and contributes to energy conservation during nutrient scarcity.

Another function of AMPK is to coordinate with the sirtuin family, particularly SIRT3 and SIRT4, to regulate mitochondrial homeostasis. Overexpression of SIRT3 in bMECs activates AMPK signaling, reduces ROS accumulation, enhances ΔΨm and ATP production, thereby protecting mitochondrial function [73]. SIRT4 modulates mitochondrial fission and fusion gene expression and mitigates ROS levels via the AMPK/mTOR axis, contributing to metabolic balance and stability of mammary gland function [74]. This synergistic regulation significantly enhances the adaptability of bMECs to metabolic stress, ensuring mammary health and lactation efficiency.

A role for AMPK in the regulation of mitophagy, at least in non-ruminants, also has been demonstrated where it activates autophagic pathways by phosphorylating ULK1 (a serine/threonine kinase) at Ser555, thereby facilitating the selective removal of damaged mitochondria [60]. In non-ruminants, a key function of ULK1 is to help the formation of the autophagosome, a key event in autophagy, a process in which at least three other proteins participate: focal adhesion kinase family interacting protein of 200 kDa (FIP200), autophagy-related protein 13 (ATG13), and ATG101 [75]. It has been reported that *Prototheca bovis* infection induces mitochondrial-dependent autophagy via the AMPK/ULK1 signaling pathway, which helps eliminate dysfunctional organelles and suppresses excessive inflammatory responses. Conversely, in periparturient ketotic cows, BHB impairs autophagy, resulting in the release of mtDNA into the cytosol and activation of the cGAS-STING inflammatory pathway. Activation of autophagy, however, can reverse this effect by removing cytosolic mtDNA and suppressing pro-inflammatory cytokine expression, thereby alleviating inflammation in bMECs [54]. These findings underscore the pivotal role of AMPK in regulating mitophagy and its potential as a therapeutic target to protect mammary tissue from inflammation-related injury.

The regulation of AMPK activity exhibits marked tissue- and context-specificity in response to varying metabolic environments. The AMPK agonist A-769662 has been shown to inhibit fatty acid synthesis by phosphorylating acetyl-CoA carboxylase at Ser79 and block nuclear translocation of sterol regulatory element-binding protein 1c (SREBP-1c), thereby reducing lipid and lactose synthesis in bMECs to maintain energy balance [76]. However, aberrant activation of AMPK may also contribute to mitochondrial dysfunction. For example, high-concentrate diets significantly increase AMPK phosphorylation and inhibit mTOR activity in the mammary gland, which promotes the expression of autophagy-related proteins (e.g., LC3II/I, Beclin1, ATG5) and reduces p62 levels, potentially aggravating oxidative stress and inducing inflammatory injury [77]. These observations suggest that while AMPK activation is critical for the control of energy metabolism, its dysregulation may disrupt mitochondrial homeostasis and exacerbate oxidative damage. By interacting with SIRT family members, mTOR, ULK1, and epigenetic modulators, AMPK participates in a dynamic regulatory network that governs mitochondrial homeostasis, offering molecular targets for nutritional interventions aimed at improving milk quality and mammary gland health.

Collectively, the mechanisms that maintain mitochondrial homeostasis in bMECs form a highly integrated and dynamically coordinated regulatory system that in the mammary gland is temporally regulated. Mitochondrial fusion and fission regulate morphology, quantity, and respond to the cellular needs for ATP, with several genes associated with these processes being activated close to and soon after calving followed by their downregulation until late-lactation. Mitophagy and the UPS ensure quality control and mitochondrial biogenesis provides functional renewal. Both AMPK and mitochondrial Ca^2+^ signaling integrate multiple signaling pathways to coordinate structural repair and energy distribution. These mechanisms operate both independently and synergistically through a complex signaling interaction network, ensuring the homeostatic function of the mammary gland under high-metabolic states such as lactation. A deeper understanding of these homeostatic regulatory mechanisms, along with their dynamic characteristics in different physiological or pathological conditions, not only helps to reveal the molecular basis of mammary gland metabolic regulation but also provides solid theoretical support and research directions for improving lactation efficiency, optimizing mammary health management, and implementing precision nutritional interventions.

## Effects of mitochondrial dysfunction on oxidative stress, apoptosis, and immune response in the bovine mammary gland

### Mitochondrial dysfunction and cellular oxidative stress

Mitochondrial ROS generation is tightly regulated by endogenous antioxidant defense systems [78]. These pathways are categorized according to the reducing molecule needed, i.e., thioredoxin (TRX)-dependent, glutaredoxin-dependent, and glutathione (GSH)-dependent [79]. In situations where ROS production exceeds the cellular antioxidant capacity, oxidative stress occurs [80]. One physiological state during which the increase in energy demands by the mammary gland to sustain milk production increases and is often accompanied by high circulating levels of ROS is the immediate period after parturition [81, 82]. In addition to the activation of metabolic processes associated with milk synthesis, the increased phosphorylation and activation of the antioxidant transcription regulator NFE2 like bZIP transcription factor 2 (NFE2L2, also known as NRF2) in mammary tissue after parturition [83] is one mechanism that helps prevent buildup of ROS in bMECs. This transcription regulator also coordinates the upregulation of GSH- and TRX-associated genes [83], which together with superoxide dismutases (SOD) help maintain redox balance.

The onset of clinical ketosis is a pathological state where the mammary gland experiences mitochondrial dysfunction and oxidative stress due to the high influx of FFA and BHB and is accompanied by an impairment of the antioxidant systems [16, 82, 84, 85]. Chen et al. [11] demonstrated that high concentrations of FFA induce mitochondrial dysfunction by increasing mtROS levels and decreasing ΔΨm, decreasing OXPHOS complexes I to V mRNA abundance and ATP levels, and suppressing mitochondrial biogenesis in bovine mammary epithelial cell line (MAC-T). These negative effects were alleviated by increased activity of NFE2L2, a response that confirmed the in vivo relevance of this antioxidant transcription regulator in the mammary gland during early lactation [59]. In addition to FFA, inflammatory molecules such as LPS also induce oxidative stress by elevating ROS and malondialdehyde (MDA) levels, and mitochondrial Ca^2+^ [10], while simultaneously impairing mitochondrial membrane integrity through suppression of antioxidant gene expression [86]. In addition to the impairment of antioxidant systems, FFA can disrupt mitophagy in mammary epithelial cells resulting in the persistent accumulation of damaged mitochondria and amplifying ROS production [18, 81, 87]. Other biologically relevant stressors for dairy cows such as HS, aflatoxin B1, the fungicide Folpet, and ammonia can induce ROS production within mammary epithelium and cause oxidative stress [88–91].

### Mitochondrial dysfunction and apoptosis

In mammalian mitochondria, excessive ROS accumulation, loss of membrane potential, and altered Ca^2+^ homeostasis all can induce the mitochondrial-mediated apoptosis pathway, which is orchestrated by increased mitochondrial outer membrane permeabilization, cytochrome c release, and caspase activation [92–94]. In light of these seemingly detrimental responses, it is important to highlight that during the transition from late-pregnancy into lactation and throughout the lactation cycle, genes associated with the release of cytochrome c from mitochondria are markedly activated in mammary gland tissue, a response that coincides with the marked activation of several pathways involving DNA damage response, mitochondrial biogenesis, caspase activation, and Ca^2+^ signaling [26]. These responses, however, are in line with the well-established balance between mammary cell proliferation and apoptosis, where there is extensive cellular apoptosis relative to proliferation during the first 60–120 d postpartum [70]. Thus, mitochondrial function not only is central for furnishing high levels of ATP during lactation but also participates in the control of mammary cell turnover.

Mounting evidence suggests that diverse stressors such as HS, *Staphylococcus aureus*, enteropathogenic *Escherichia coli* (EPEC), and the pesticide pyridaben can induce mammary epithelial cell apoptosis by impairing mitochondrial function and reducing milk production and quality [95–99]. Of note, Sun et al. reported that targeting NRF2 can alleviate H_2_O_2_-induced apoptosis, likely because NRF2 activation enhances the expression of antioxidant enzymes, reduces mtROS accumulation, and protects mitochondrial structure and function [98]. Additionally, NRF2 activation up-regulates the expression of PGC-1α, thereby facilitating the biogenesis of new mitochondria [98].

The dynamin-related protein 1 (DRP1) protein is a key molecule in mitochondrial fission and has been highlighted in related studies. Li et al. reported that in MAC-T treated with EPEC there was increased mitochondrial fission, decreased fusion, and decreased ΔΨm all of which contributed to apoptosis. The pharmacological inhibition of mitochondrial fission mitigated EPEC-induced apoptosis, and the mitochondrial morphology could be partially restored [96]. The protein UFBP1 (Ufm1-binding protein 1) plays a pivotal role in the process of UFMylation, which is essential for maintaining cellular homeostasis. It was reported that the mitochondrial apoptosis pathway triggered by HS was blocked by UFBP1. This was evidenced by the increase in ΔΨm, enhanced ATP synthesis, and an elevated NAD ^+^/NADH ratio, as well as a reduction in ROS generation [100]. These findings collectively indicate that maintaining mitochondrial homeostasis through targeted regulation of key molecules such as NRF2 and DRP1 is a potential strategy to mitigate apoptosis in mammary epithelial cells under pathological conditions.

### Mitochondrial dysfunction and innate immunity

Mounting evidence suggests that mitochondria are a critical signaling hub in innate immune pathways. In addition to regulating antiviral or antibacterial immunity, mitochondria are also key drivers of inflammation caused by sterile injury [101–103].

#### Toll-like receptors (TLRs)

The TLRs belong to a family of trans-membrane pattern recognition receptors (PRR) that recognize inflammatory molecules, can sense tissue damage, and can recognize pathogens to trigger an immune response [104, 105]. For example, in bovine mastitis induced by *Staphylococcus aureus* infection, TLR2 and TLR4 are the predominant TLR members expressed [106] and their activation initiates the NF-κB/MAPK pathways, which contribute to the release of pro-inflammatory cytokines [106]. Under homeostatic physiological conditions, mtDNA is strictly confined to the mitochondrial matrix, effectively isolated from cytosolic PRRs. However, mitochondrial stress or damage can compromise this compartmentalization, resulting in mtDNA leakage into the cytosol [107].

The fact that mammary gland tissue of Holstein cows had marked activation of genes associated with the release of cytochrome c from mitochondria from late-pregnancy (d −15 prepartum) through most of lactation suggests that release of mtDNA into the cytosol is a normal event, facilitated by pore formation in the mitochondrial membrane [108]. In non-ruminants undergoing pathologies that lead to inflammation, the release of mtDNA is recognized as a key response of the mitochondria [107, 109], where the increase in cytosolic mtDNA amplifies inflammation via TLR9 and can lead to tissue damage. In lactating dairy cows, metabolic disorders associated with energy balance such as ketosis or infectious diseases such as mastitis increase mtDNA abundance in mammary tissue, serum and milk [54, 110]. Although a mammary-specific disease such as mastitis leads to activation of the TLR2/TLR4 and NF-κB/MAPK pathways and mtDNA release [106], in the course of a normal lactation, the activation of IκB kinase/NF-κB signaling [26] is not necessarily due to TLR upregulation, and could arise from the production of molecules such as anaphylatoxins, opsonins, or the terminal membrane attack complex, all of which compose the complement cascade which is activated during lactation [26]. Clearly, in order to sustain not only the metabolic needs of lactating mammary cells, there are mechanisms that tie together the energy-generating function of the mitochondria, the release of bioactive molecules (cytochrome c, mtDNA), and immune-related pathways with metabolic and non-metabolic events all of which are normal responses in these cells as lactation progresses.

#### NF-κB signaling and NLRP3 inflammasome

The transcription factor NF-κB, a key activator of inflammation, primes the NLRP3-inflammasome for activation through transcriptional up-regulation of the expression of both pro-IL-1β and NLRP3 [111]. The NLRP3 inflammasome is a multi-protein complex that consists of pro-Caspase-1, apoptosis associated spot like protein (ASC), and NLRP3 [112]. Under stressful stimuli, the NLRP3 inflammasome is activated and assembles into a functional complex, which subsequently promotes the maturation and secretion of the pro-inflammatory cytokines IL-1β and IL-18 [93]. Through several distinct pathways, in particular mitochondrial dysfunction, the mitochondria are critical regulators of NLRP3 inflammasome activation [93]. These interconnected pathways include: mtROS production, Ca^2+^ overload, NAD ^+^ levels, cardiolipin externalization to the outer membrane, MFN1 and MFN2, release of mtDNA, and decreased mitochondrial antiviral signaling protein (MAVS) [113].

Although after parturition the dairy cow is exposed to transient increases in circulating concentrations of FFA due to NEB [82, 114, 115], under normal conditions, the FFA are transported through the bloodstream to the liver where they are metabolized to ATP, CO_2_, or ketone bodies, or to the mammary gland where they are used primarily for milk fat synthesis [116].

The process of acetylation of lysine residues in histone proteins opens the chromatin structure and allows binding of RNA polymerase II and transcription regulators to DNA sequences, triggering gene transcription [117]. The histones deacetylation represses gene transcription by blocking transcriptional machinery to the DNA. SIRT3, a member of the class III NAD^+^-dependent enzymes, deacetylates and activates mitochondrial acetyl-CoA synthetase (ACSS2). As a downstream target gene of *PPARGC1A*, it plays crucial roles in mitochondrial biogenesis and function [3, 118]. Although there are no published data, to our knowledge, of the expression patterns of the sirtuins in the bovine mammary gland during lactation, if PGC-1α in the bovine controls’ transcription of SIRT3 it seems feasible that an upregulation of *PPARGC1A* also could trigger upregulation of *SIRT3*. Thus, the fact *PPARGC1A* and *ACSS2* mRNA abundance was markedly upregulated in the mammary gland during lactation [25] is indicative of an important role for SIRT3.

In the context of pathological conditions, there is evidence from non-ruminant studies that overt inflammation can inhibit *PPARGC1A* transcription and function in organs like the kidney and pancreas [119, 120]. In bMECs, the increase in both LPS or FFA exposure was associated with downregulation of SIRT3 and PPARGC1A in part due to activation of NF-κB signaling [121, 122]. These in vitro studies also highlighted that overexpression of SIRT3 attenuated LPS-induced inflammatory responses by regulating the PGC-1α/NF-κB signaling pathway [122], hence, providing a potential therapeutic target for preventing or treating inflammation in dairy cows.

#### cGAS-STING pathway

The cGAS-STING signaling pathway consists of cGAS, STING, and downstream signaling adaptor, which is activated by mitochondrial dysfunction [123]. It induces an innate immune response by detecting microbial DNA and internal damage-associated DNA [123]. When cells are invaded by microorganisms or exposed to danger signals, mitochondrial structure and function become abnormal, releasing mtDNA into the cytosol [103], an effect that has been reported in serum and milk of dairy cows with mastitis [110]. Invading bacteria also produce cyclic dinucleotides (c-di-GMP, c-di-AMP, cGAMP) which along with mtDNA can bind to cGAS, activate the STING pathway, and the TANK binding kinase 1/interferon regulatory factor (IRF) phosphorylation cascade leading to production of type I interferons and the transcription of pro-inflammatory genes [103]. Metabolic disorders such as diabetes and obesity in non-ruminants are associated with activation of the cGAS-STING signaling pathway [124, 125]. Similarly, metabolic disorders like ketosis in dairy cows lead to increased cytoplasmic mtDNA abundance and activation of the cGAS-STING pathway in mammary tissue [54].

After parturition, although the mammary gland experiences a modest upregulation of IRF6 and IRF7, there is marked downregulation of unc-51 like autophagy activating kinase 2 (ULK2) [26], which upon translation is involved in the phosphorylation of STING, a crucial step of the STING-mediated interferon response. Thus, it seems that in healthy cows the STING pathway is not a major driver of the inflammatory response controlled by the activation of the IκB kinase/NF-κB cascade [26].

In summary, mitochondrial dysfunction is closely related to the imbalance of mammary tissue homeostasis. Recently, several studies have demonstrated that natural products can protect the udder health and improve milk production by maintaining mitochondrial fitness (Table 1).

**Table 1:** Herbs targeting mitochondria in mammary epithelial cells of dairy cows

Targets	Herbs	Detection indicator	In vitro	References
Mitochondrial biogenesis	Resveratrol	↑PGC-1α, ↑Mitochondrial biogenesis-related genes	In LPS-treated bMECs	[118]
ROS	Curcumin	↓ROS,↓MDA ↑SOD, ↑GSH	In LPS-treated bMECs	[126]
	Baicalin	↓ROS	In H_2_O_2_-treated bMECs	[127, 128]
	Lycopene	↓ROS ↑GSH,↑SOD	In bMECs treated with H_2_O_2_	[98]
	Tea polyphenols	↓ROS	In bMECs with H_2_O_2_	[129]
	Hesperidin	↓ROS, ↓MDA, ↑CAT	In bMECs challenged with H_2_O_2_	[130]
	Resveratrol	↓ROS	In bMECs induced by aflatoxin B1 or H_2_O_2_	[118, 131]
	Tanshinone IIa	↑Activities of antioxidant enzymes	In LPS-challenged bMECs	[132]
	Puerarin	↓MDA,↓ROS,↑ SOD,↑GSH, ↑CAT	In bMECs challenged with H_2_O_2_	[133]
Inflammatory response	Allicin	↓IL-1β,↓IL-6,↓IL-8, ↓TNF-α,↓NLRP3 inflammasome	In bMECs challenged with LPS	[134]
	Curcumin	↓TNF-α, ↓IL-8, ↓IL-6 and ↓IL-1β	In bMECs challenged with LPS	[126]
	Baicalin	↓NF-κB signaling, ↓TNF-α, ↓IL-8, ↓IL-6 and ↓IL-1β	In bMECs challenged with LPS	[128]
	Quercetin	↓TNF-α, ↓IL-1β,↓IL-6	In LPS-challenged bMECs	[135]

### Inducing mitochondrial biogenesis

The number of mitochondria is a critical factor in mitochondrial function. Mitochondrial biogenesis is a tightly regulated process in which new mitochondria are generated from pre-existing mitochondria to increase the mitochondrial numbers [136]. Pharmacological induction of mitochondrial biogenesis may be a therapeutic approach to mitigate mitochondrial dysfunction. For example, resveratrol is a natural plant polyphenol that is abundant in grapes, blueberries, nuts, and other plants [118, 137]. Hu et al. reported that the abundance of *PPARGC1A* and mitochondrial biogenesis-related genes was down-regulated in lactating dairy cows with mastitis or in bMECs exposed to LPS. However, in LPS-treated bMECs, resveratrol ameliorated mitochondrial damage by up-regulating the abundance of *PPARGC1A* and the mitochondrial biogenesis-related genes [118].

### Preventing oxidative stress

Mitochondria generate the majority of cellular ROS in mammalian cells [138]. Excessive ROS accumulation overwhelms cellular antioxidant defense and leads to oxidative stress, which contributes to cellular dysfunction and the development of various pathological conditions [139]. Increasing mitochondrial antioxidant defenses can rapidly remove excess ROS, and maintain cellular homeostasis [139]. Moreover, excessive mtROS activates the NLRP3 inflammasome pathway, which amplifies inflammation and damage to mammary tissue [18]. Accumulating evidence suggests that mammary tissue from cows experiencing ketosis, HS, and mastitis also experience oxidative stress and inflammatory response, which likely contributes to impaired milk production and quality [12, 16, 82, 140]. Thus, there is ongoing interest in finding novel approaches to prevent or reduce the risk of exposure to prolonged periods of oxidative stress.

Among the nutritional approaches being studied are the various polyphenols that can be found in feeds. These molecules can trigger antioxidant responses through the activation of the NFE2L2 pathway, which was demonstrated to be active in the bovine mammary gland in vivo [59]. Curcumin, an active component of turmeric, reduced ROS levels and MDA content and improved the activity of SOD and GSH induced by LPS in bMECs [126], and it also attenuated the expression of TNF-α, IL-8, IL-6 and IL-1β via inactivation of NF-κB signaling [126]. Pretreatment with baicalin, the main bioactive compound in *Scutellaria* roots, reduced H_2_O_2_-induced ROS production in bMECs [127]. As well, it ameliorated LPS-induced inflammation in bMECs via inhibition of NF-κB activation and up-regulation of HSP72 [128]. Lycopene, a naturally occurring hydrocarbon carotenoid [98], mitigated ROS production and elevated the level of GSH and SOD in bMECs treated with H_2_O_2_ via the NFE2L2 pathway. Tea polyphenols, the main polyphenolic constituents of green tea [129, 141], work through the activation of the NFE2L2/HMOX1 pathway to decrease ROS levels induced by incubation of bMECs with H_2_O_2_ [129]. Puerarin, a flavonoid extracted from the plant *Pueraria*, reduced the MDA content and ROS levels, and enhanced the activities of SOD, GSH, and CAT in bMECs challenged with H_2_O_2_ [133]. Hesperidin is a flavonoid in citrus fruits such as oranges and lemons [130], that reduced the levels of ROS and MDA and effectively ameliorated the reduction in CAT via activation of the Keap1/NRF2/ARE signaling pathway in bMECs challenged with H_2_O_2_ [130]. In addition, resveratrol alleviates an increase in ROS in bMECs induced by aflatoxin B1 or H_2_O_2_ through the NFE2L2 signaling pathway [131, 137]. Tanshinone IIa is a diterpenoid extracted from *Salvia miltiorrhiza* [132] that improved the activities of antioxidant enzymes in LPS-challenged bMECs by activating the Keap1/NRF2 pathway [132]. Quercetin, a flavonoid found in many fruits and vegetables [142], down-regulated the abundance of the pro-inflammatory cytokines TNF-α, IL-1β and IL-6 via suppression of the TLR4-mediated NF-κB signaling pathway in LPS-challenged bMECs [135]. Allicin, an active Sulphur compound found in garlic, onions and other alliaceous plants, reduced the LPS-induced increase in the levels of the inflammatory cytokines IL-1β, IL-6, IL-8 and TNF-α and inhibited the activation of the NLRP3 inflammasome in bMECs [134].

Although the aforementioned traditional Chinese medicine ingredients exhibit significant antioxidant and anti-inflammatory activities at the cellular level, their effects have not yet been further verified by in vivo experimental data. The actual protective efficacy, potential side effects, and details of the mechanism of action of these natural products on mammary epithelial cells still need to be clarified.

## Conclusions

Metabolic stress or pathogenic infections can disrupt mitochondrial homeostasis in dairy cow mammary tissue leading to oxidative stress, inflammatory damage, and apoptosis, ultimately affecting lactation success. Thus, targeting the mitochondria might be a therapeutic strategy for alleviating dysfunction of the mammary gland. Despite the substantial amount of published research on several aspects related to mitochondria and mammary function, there is still a need to better understand the physiological roles of this organelle in order to identify key intracellular targets that could help improve tissue homeostasis. In that context, several herbal medicine-based therapeutic strategies targeting mitochondrial pathways could offer solutions, but clinical trials need to be performed to ascertain their effects in vivo.

## Data Availability

Not applicable.

## Abbreviations

AMPK: AMP-activated protein kinase
ASC: Apoptosis associated spot like protein
ARE: Antioxidant response element
ATG13: Autophagy-related protein 13
BHB: β-Hydroxybutyrate
bMECs: Bovine mammary epithelial cells
CAT: Catalase
CASTOR1: Cytosolic arginine sensor for mTORC1 subunit 1
cGAS: Cyclic GMP-AMP synthase
CREB: CAMP responsive element binding proteins
DRP1: Dynamin-related protein 1
DNMT1: DNA methyltransferases 1
EZH2: Enhancer of zeste homolog 2
ERRα: Estrogen-related receptor alpha
MDA: Malondialdehyde
MECs: Mammary epithelial cells
EPEC: Enteropathogenic *Escherichia coli*
ER: Endoplasmic reticulum
FIP200: Focal adhesion kinase family interacting protein of 200 kDa
FIS1: Fission 1
GSH: Glutathione
HMOX1: Heme oxygenase 1
HS: Heat stress
LPS: Lipopolysaccharide
MAMs: Mitochondria-associated ER membranes
MCUR: Mitochondrial calcium uniporters
MDV: Mitochondrial-derived vesicles
MFN1/2: Mitofusin 1/2
mTORC1: Mechanistic target of rapamycin (mTOR) complex 1
mtROS: Mitochondrial reactive oxygen species
MUL1: Mitochondrial E3 ubiquitin protein ligase 1
NFE2L2: NFE2 like bZIP transcription factor 2
NRF1/2: Nuclear respiratory factor 1 and 2
OPA1: Optic atrophy 1
OXPHOS: Oxidative phosphorylation
PINK1: PTEN-induced putative kinase 1
PPARGC1A: Peroxisome proliferator-activated receptor gamma coactivator 1-alpha
ROS: Reactive oxygen species
SENP1: SUMO specific peptidase 1
SIRT1: Sirtuin 1
SOD: Superoxide dismutase
SREBP-1c: Sterol regulatory element-binding protein 1c
STING: Stimulator of interferon genes
TAX1BP1: Tax1 binding protein 1
TOLLIP: Toll interacting protein
TRX: Thioredoxin
UFBP1: Ufm1-binding protein 1
ULK2: Unc-51 like autophagy activating kinase 2
UPS: Ubiquitin–proteasome system
VDAC: Voltage-dependent anion-selective channels
